# Lung and gut microbiota profiling in severe community acquired pneumonia patients: a prospective pilot study

**DOI:** 10.3389/fmicb.2025.1717822

**Published:** 2025-11-27

**Authors:** Wei Zou, Ruobin Zheng, Sheng Lin, Chunhua Lu, Baosong Xie

**Affiliations:** 1 Department of Respiratory and Critical Care Medicine, Fuzhou University Affiliated Provincial Hospital, School of Medicine, Fuzhou University, Shengli Clinical Medical College of Fujian Medical University, Fuzhou, China; 2 Department of Respiratory and Critical Care Medicine, Fuzhou Changle District People's Hospital, Fuzhou, China

**Keywords:** gut, lung, microbiomes, severe community-acquired pneumonia, 16S rRNA gene sequencing

## Abstract

**Background:**

The gut and lung microbiomes play crucial roles in host defense and may serve as predictive markers for severe community-acquired pneumonia (SCAP) patients. However, the simultaneous landscapes of lung and gut microbiomes for SCAP patients remain unclear. The primary objective of this research is to investigate the concomitant landscape of the lung and gut microbiota between the death group and the survival group of SCAP patients and to identify microbial features predictive of clinical parameters.

**Methods:**

We analyzed 50 respiratory samples and 50 stool samples collected from 50 SCAP patients in this prospective observational study. Patients were categorized into the survival group (*n* = 41) and the death group (*n* = 9) according to clinical outcomes. We characterized microbiome compositions, LEfSe analysis, UPGMA analysis and correlation of microbiota features with clinical parameters of respiratory and intestinal flora between two groups using 16S rRNA gene sequencing.

**Results:**

In comparison with the survival group, the death group demonstrated a reduction in alpha diversity, most markedly reflected in the lung microbiota. We found enrichment of specific lung bacterial taxa (Bacteroidales, Streptococcus) in the survival group compared to the death group. Similarly, specific gut bacterial taxa (Anaerotruncus, Peptacetobacter, Rutheniibacterium) were also enriched in the survival group Our study revealed that lung bacteria such as Asteroleplasma, Campylobacter and Acinetobacter and intestinal bacteria such as Bifidobacterium, Ligilactobacillus, Veillonella, and Corynebacterium were positively correlated with inflammatory markers PCT or CRP or neutrophil percentage. Besides, lung bacteria such as Schaalia and intestinal bacteria Alistipes were positively correlated with PaO_2_/FiO_2_, while lung bacteria such as Stenotrophomonas was negatively correlated with PaO_2_/FiO_2_.

**Conclusions:**

Our findings reveal distinctive microbial profiles in lung and gut microbiota that correlate with clinical outcomes in SCAP patients. Unraveling these microbial patterns could enable targeted interventions to improve outcomes of SCAP patients.

## 1 Background

Severe community-acquired pneumonia (SCAP) constitutes a critical public health issue due to its high incidence and mortality rates. Globally, the incidence of community-acquired pneumonia (CAP) ranges from 27 to 88 cases per 100,000 people, with approximately 5–35% of these CAP cases progressing to SCAP, resulting in an in-hospital mortality rate of 30–50% (Ferrer et al., 2018; Liu et al., 2023). The interplay between the lung and gut microbiomes—defined as the gut-lung axis—is increasingly recognized as a critical regulator of the host's overall health and immune response, particularly in patients with SCAP. This interaction is crucial for maintaining immunological homeostasis, and its disruption can significantly influence the progression and severity of SCAP (Harris and Marsland, 2025; He et al., 2017).

The alteration of the lung microbiome, particularly evident in SARS-CoV-2 infections (Cyprian et al., 2021), correlates with disease progression by inducing profound inflammatory reactions and disrupting immune mechanisms (Drigot and Clark, 2024), significantly affecting alveolar macrophages that are essential for pathogen clearance (Baasch et al., 2021). Gut dysbiosis, an imbalance in the gut microbial community, can exacerbate the severity of respiratory infections and affect the efficacy of treatments. Short-chain fatty acids (SCFAs) produced by the gut microbiome can inhibit lung inflammation by activating G protein-coupled receptors, indicating that metabolites from the gut microbiome play a crucial role in modulating pulmonary immune responses (Ashique et al., 2022; Dang and Marsland, 2019). Moreover, gut microbiome dysbiosis is closely associated with the severity of lung infections. In patients with SCAP, gut microbiota diversity is significantly reduced, which is linked to the exacerbation of pulmonary inflammation (Zeng et al., 2023). Studies have further revealed that gut microbiome imbalance can aggravate lung damage by affecting the balance of Th1/Th2 cells (Yang et al., 2022).

Dysbiosis in lung and gut microbiomes may lead to SCAP, impacting patient outcomes and survival. Yet, the comprehension of relationships between lung and gut microbiota and host vulnerability in severe pneumonia remains inadequate. Prior investigations have mainly centered on either lung or gut microbiota, with scarce research simultaneously assessing both compartments, specifically for SCAP patients (Dickson et al., 2020; Schlechte et al., 2023). Furthermore, the relationship between microbiota profiling and clinical outcomes, such as survival and death, is not thoroughly studied in the SCAP patients. To date, no study has determined whether altered lung and gut microbiota predict death in this population. Therefore, this study aims to comprehensively profile lung and gut microbiota between the death group and the survival group of SCAP. By elucidating the associations between lung-gut microbiota dynamics and in-hospital mortality, we seek to provide novel insights into the pathogenesis and potential therapeutic strategies for SCAP.

## 2 Methods

### 2.1 Study population

This is a prospective single-center study of SCAP patients. The study was conducted in the Fuzhou University Affiliated Provincial Hospital between January 2024 to January 2025. Our study population comprised all patients over 18 years of age with SCAP diagnosis according to the Infectious Diseases Society of America (IDSA)/American Thoracic Society (ATS) guidelines (Metlay et al., 2019). Exclusion criteria were: (1) conditions, such as hospital-acquired pneumonia, tuberculosis, novel coronavirus infection, (2) immunocompromised, (3) antibiotic treatment in the last 30 days, (4) inability to obtain intestinal or respiratory specimens during the prospective study. The definition of immunocompromised status aligns with the most recent scholarly consensus published in Chest journal in 2020 (Ramirez et al., 2020). In total, 50 patients were enrolled finally for further detailed analysis by inclusion/exclusion criteria in the study and categorized into the survival group and death group according to clinical outcomes. Ethical approval for this research has been obtained from the Ethics Committee of Fuzhou University Affiliated Provincial Hospital (Ethical Review No. K2023-08-018). Additionally, informed consent was secured from each participant.

### 2.2 Specimen collection

Bronchoalveolar lavage fluid (BALF) Collection: Bronchoalveolar lavage fluid (BALF) was collected within 48 h of patient admission through a standardized bronchoscopic technique performed by skilled pulmonary specialists. Guided by chest computed tomography imaging, bronchoscopists carefully navigated the endoscope to the affected lung segment, maintaining strict sterile protocol. Sterile physiological saline was gently instilled into the targeted bronchial region and subsequently retrieved using controlled negative pressure aspiration.

#### 2.2.1 Sputum collection

To minimize oral contamination, patients were instructed to thoroughly rinse their mouth 3–5 times before expectoration and then to expectorate sputum forcefully into a designated collection cup. microscopic examination to verify sample quality (>25 neutrophils and < 10 squamous epithelial cells per low-power field), exclusion of samples failing quality criteria, and bioinformatics filtering of typical oral flora contaminants.

#### 2.2.2 Fecal sample collection

Fecal samples were collected within 48 h of patient hospitalization. After hand hygiene and donning sterile gloves, the internal mid-portion of the fecal sample was collected using a sampling spoon from the fecal collection tube, obtaining approximately 0.5–1.0 grams (including surface intestinal mucosal cells). The sample was then transferred to a cryogenic tube, labeled, and coded.

Respiratory and fecal samples were collected from enrolled patients, rapidly frozen, and stored in liquid nitrogen at −80°C until use. All collected samples were systematically grouped and collectively sent to Shenzhen BGI Genomics Company for comprehensive 16S rRNA sequencing analysis.

### 2.3 Microbial DNA extraction, library preparation, and sequencing

Negative (blank) extraction controls processed in parallel with clinical samples to monitor potential contamination; these controls were sequenced and analyzed alongside study samples, and no significant microbial signal was detected. DNA Extraction Procedure Using Magnetic Bead Method for Samples (Sputum, Bronchoalveolar Lavage Fluid, Fecal Specimens): A 100–200 mg sample was combined with grinding beads and 1 mL of Buffer ATL/PVP-10, then subjected to high-speed homogenization. The mixture was subsequently incubated at 65°C for 20 min to facilitate cell lysis. After centrifugation at 14,000 g for 5 min, the supernatant was collected and mixed with 0.6 mL Buffer PCI, followed by another centrifugation (18,213 g for 10 min). The resulting supernatant was transferred to a deep-well plate pre-loaded with magnetic bead binding solution (600 μL), Proteinase K (20 μL), and RNase A (5 μL). DNA purification was performed using the Kingfisher automated workstation, with final elution of DNA using 100 μL of elution buffer. The extracted genomic DNA was used to construct a metagenomic amplicon library. Fusion primers were designed to include sequencing primers and conserved regions, and PCR amplification was performed targeting the variable regions of 16S rRNA gene. After passing quality control, the amplification products underwent circularization, forming single-stranded circular DNA. These circular DNAs were then subjected to rolling circle amplification to generate DNA nanoballs (DNBs). Following extraction, researchers meticulously prepare sequencing libraries by amplifying specific 16S rRNA gene hypervariable regions (typically V3–V4), attaching unique molecular identifiers and sequencing adapters. The prepared libraries are then loaded onto high-throughput sequencing platforms like Illumina NovaSeq, generating millions of paired-end reads. Subsequent bioinformatics analysis involves rigorous quality filtering, host DNA removal, taxonomic classification, and clustering of reads into operational taxonomic units, ultimately producing comprehensive microbial composition profiles that reveal the intricate microbial landscape of the original samples. The V3–V4 hypervariable regions of the 16S rRNA gene were amplified using universal primers. Following quality control, the median sequencing depth was 45,823 reads per sample for respiratory specimens (range: 32,156–68,492) and 52,347 reads per sample for fecal specimens (range: 38,921–72,156). To account for variations in sequencing depth and ensure comparability across samples, all samples were rarefied to the minimum library size (30,000 reads) prior to alpha diversity calculations using the “rarefy_even_depth” function in the phyloseq package.

### 2.4 Statistical analysis

The bioinformatics analysis began with Cutadapt (v2.6) for primer and adapter sequence removal, implementing rigorous quality control by eliminating low-quality reads, N-base-containing sequences, and low-complexity reads. Paired-end sequences were assembled using FLASH (v1.2.11), followed by OTU clustering at 97% similarity threshold with USEARCH (v7.0.1090) and chimeric sequence removal via UCHIME (v4.2.40). Taxonomic classification was performed using RDP classifier (v2.2) with a 0.6 confidence threshold, enabling precise microbial identification. Comprehensive diversity analyses included Alpha diversity indices (Chao1 and Shannon) calculated through mothur (v1.31.2) and Beta diversity assessment using QIIME (v1.80) and microbiota relative abundance comparisons using Spearman correlation analysis. An LDA threshold of 2.0 was used for the LEfSe analysis to identify significant microbial taxa.

Data analysis was performed using SPSS 29.0 statistical software. Normally distributed quantitative variables were represented by mean ± standard deviation, non-normally distributed variables by median [interquartile range], and categorical variables by count and percentage. Comparative analyses between groups employed independent samples *t*-test for normally distributed continuous variables, Mann-Whitney U test for non-normally distributed continuous variables, and chi-square or Fisher's exact test for categorical variables, ensuring a comprehensive and statistically rigorous approach to data interpretation and hypothesis testing.

## 3 Results

### 3.1 Patients' characteristics

**Figure 1:**
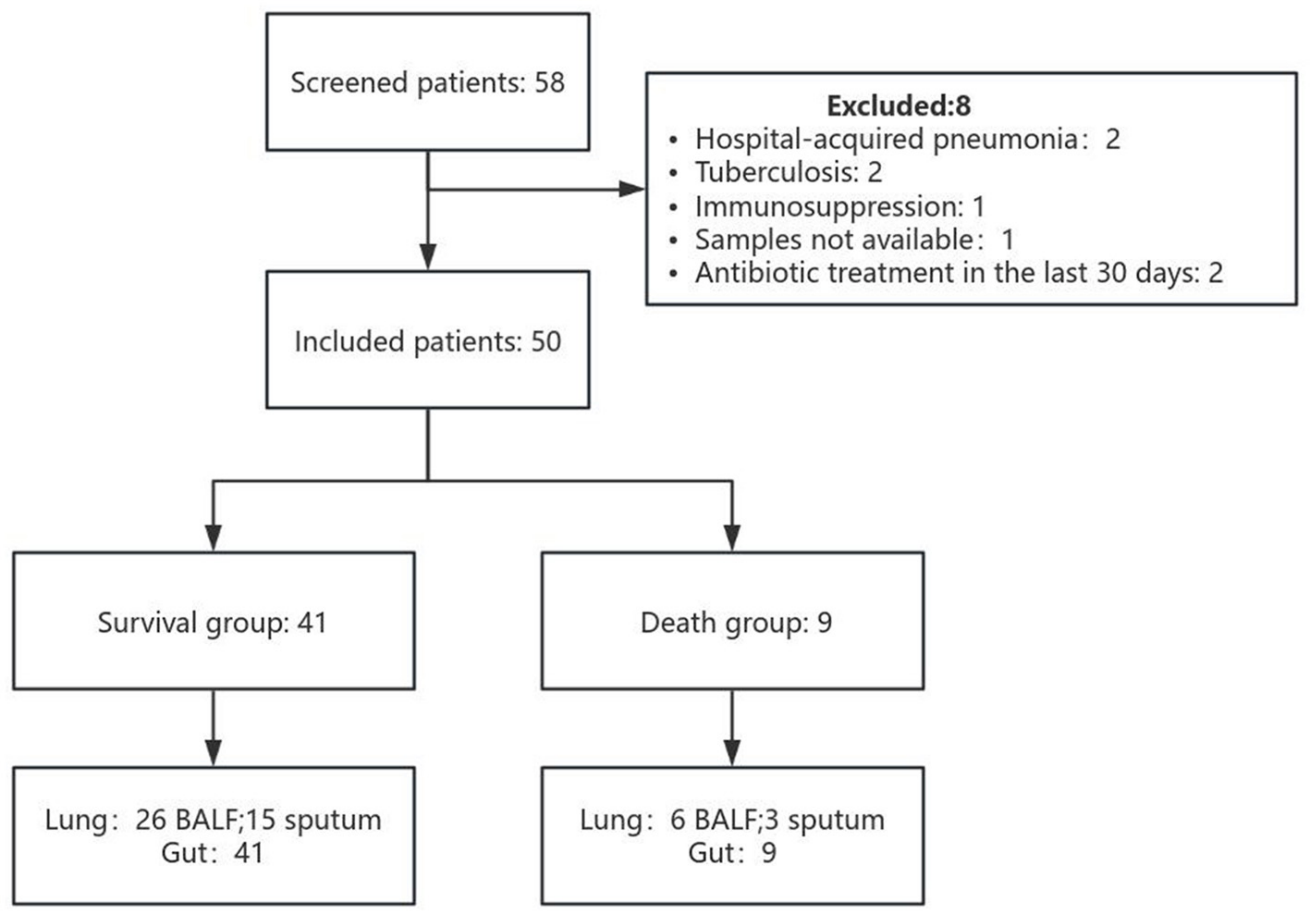
Scheme of study participants and sample collection. Lung samples refer to BALF and sputum; gut samples refer to stool.

Among all prospectively screened patients, fifty-eight patients fulfilled the inclusion criteria. Eight patients were secondarily excluded as shown in the study flowchart (Figure 1). Among the remaining 50 patients, 9 (18%) patients died (the death group) and 41 (82%) patients survived (the survival group). In total, 50 respiratory samples and 50 stool samples were collected for microbiome evaluation. Of the 50 respiratory samples collected, 32 were BALF samples and 18 were sputum samples. In the survival group, 26 BALF and 15 sputum samples were collected; in the death group, 6 BALF and 3 sputum samples were obtained. A total of 50 patients were included in this study finally, among whom 41 (82%) survived and 9 (18%) died. Patient characteristics and baseline were shown in Table 1. In the survival group, 78% (32 out of 41) of patients were male, and the mean age was 49.5. In the death group, 77.8% (7 out of 9) of patients were male, and the mean age was 75. Baseline characteristics did not significantly differ between the two groups of patients besides the number of cases with mechanical ventilation and sepsis as a complication and the level of the APTT, which were higher in the death group than in the survival group (p < 0.05).

**Table 1:** Patient characteristics and baseline of the two groups.

**Variable**	**Survival group *n* (%)**	**Death group *n* (%)**	***P-*value**
**Characteristics**			
**Gender**			
Male	32 (78.00)	7 (77.80)	0.986
Female	9 (22.00)	2 (22.20)	
Age	69.46 ± 13.52	75.00 ± 7.50	0.284
Length of hospital stay	21 (16–29)	25 (5–48)	0.990
BMI	21.80 ± 2.60	21.80 ± 1.700	0.230
Smoking history	16 (39.00)	2 (22.20)	0.342
Drinking history	6 (14.60)	0 (0)	0.221
**Hospitalized status**			
ICU	26 (63.40)	8 (88.90)	0.138
General ward	15 (36.60)	1 (11.10)	
**Method**			
Non-mechanical ventilation	26 (63.40)	1 (11.10)	0.004^*^
Mechanical ventilation	15 (36.60)	8 (88.90)	
**Underlying diseases**			
Hypertension	30 (73.20)	8 (88.90)	0.425
Diabetes	13 (31.70)	3 (22.20)	0.705
Coronary heart disease	10 (24.40)	3 (33.30)	0.679
Cerebral infarction	12 (29.30)	2 (22.20)	1
Parkinson's disease	6 (14.60)	0 (0)	0.576
COPD	4 (9.80)	0 (0)	1
Asthma	2 (4.90)	0 (0)	1
Interstitial lung disease	7 (17.10)	3 (33.30)	0.358
Arrhythmia	12 (29.30)	4 (44.40)	0.442
**Complications**			
Sepsis	11 (26.80)	7 (77.80)	0.007^*^
Acute kidney injury	11 (26.80)	5 (55.60)	0.124
Acute liver failure	18 (43.90)	6 (66.70)	0.281
Respiratory failure	32 (78.00)	8 (88.90)	0.665
ARDS	1 (2.400)	2 (22.20)	0.080
**Laboratory parameters**			
WBC (×10^∧^9/L)	11.32 ± 4.92	16.24 ± 10.40	0.200
Hb (g/L)	106.88 ± 25.30	103.11 ± 22.98	0.683
PLT (×10^∧^9/L)	254.90 ± 146.42	185.78 ± 114.38	0.191
PCT (ug/L)	1.40 (0.32–3.76)	1.45 (0.44–9.74)	0.561
CRP (mg/L)	137.83 ± 92.60	157.98 ± 72.48	0.565
APTT (s)	33.55 (29.01–36.55)	44.90 (34.30–49.30)	0.015^*^
D-D (mg/L)	2.87 (1.56–5.11)	4.3 (2.8–5.82)	0.101
Alb (g/L)	30.69 ± 4.39	29.44 ± 1.67	0.168
BUN (mmol/L)	11.28 ± 6.46	15.69 ± 11.68	0.302
CREA (mmol/L)	94 (63.75–146.50)	114 (76.5–358)	0.306
PaO_2_/FiO_2_(mmHg)	168.71 ± 53.87	130.23 ± 45.33	0.066
Lac (mmol/L)	1.67 ± 0.85	2.08 ± 0.75	0.211

BMI, Body Mass Index; ICU, Intensive Care Unit; COPD, Chronic Obstructive Pulmonary Disease; ARDS, Acute Respiratory Distress Syndrome; WBC, white blood cell; Hb, hemoglobin; PLT, platelet count; PCT, procalcitonin; CRP, C-reactive protein; APTT, activated partial thromboplastin time; Alb, albumin; BUN, Blood Urea Nitrogen; CREA, Creatinine; PaO_2_/FiO_2_, Oxygenation Index; Lac, Lactic Acid.

^*^p < 0.05.

### 3.2 Respiratory and gut microbiota landscapes between the death group and the survival group of SCAP patients

**Figure 2:**
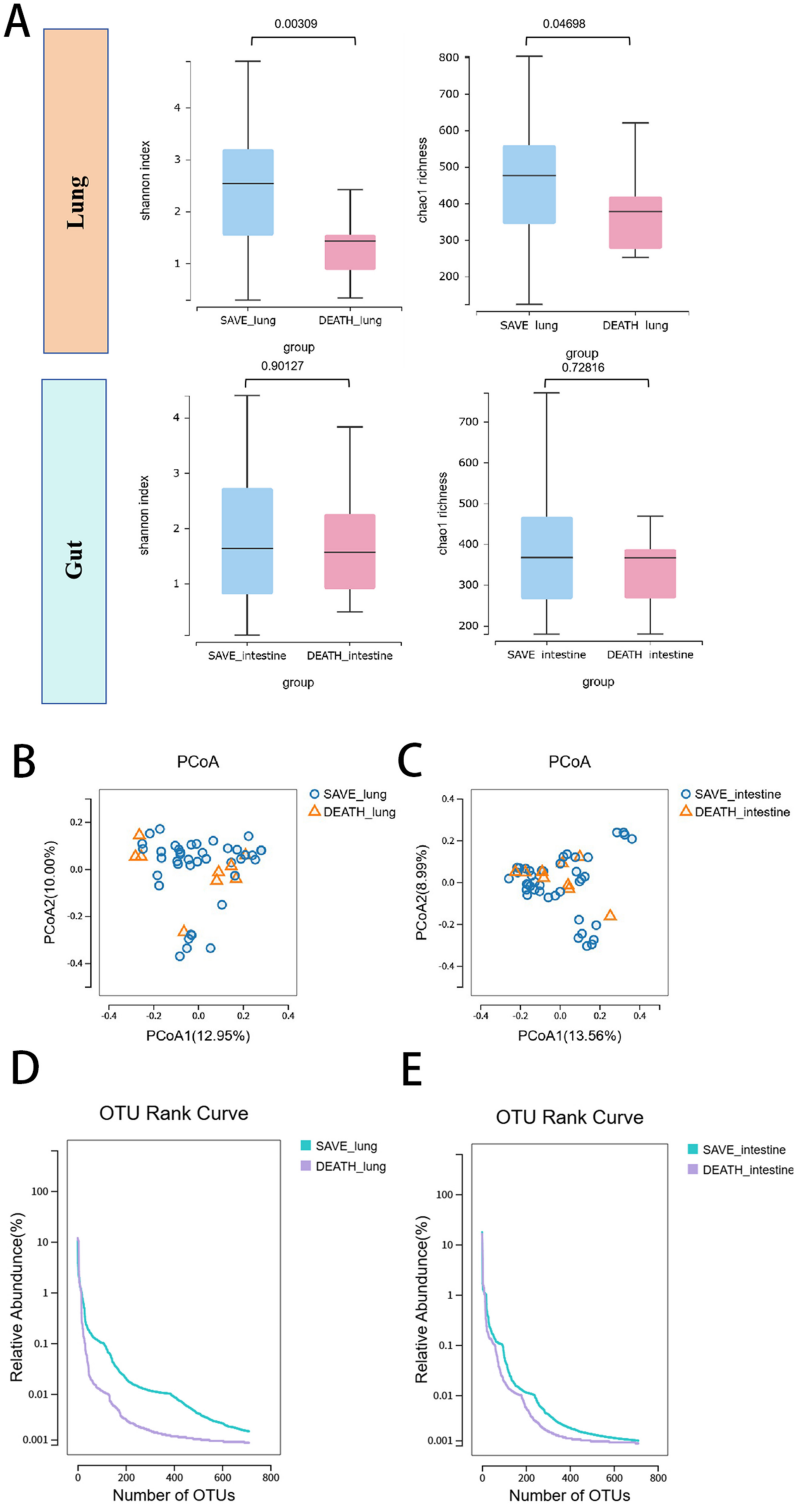
Respiratory and gut microbiota landscapes between the death group and the survival group of SCAP patients. **(A)** Lung and gut microbial richness based on the different alpha-diversity index between the death group and the survival group. The box represented the interquartile range (IQR) and the midline represented the median. The PCoA of **(B)** lung and **(C)** gut microbial species level between two groups. OTU rank curves were generated to illustrate the **(D)** respiratory and **(E)** gut microbial community structure between two groups. OTUs, operational taxonomic units.

We first evaluate alpha-diversity within lung and gut microbial communities between the death group and the survival group of SCAP patients. As was shown in Figure 2A, alpha diversity differs significantly between the two groups as expressed by Shannon or Chao1 diversity index for the lung microbiome (*p* = 0.00309 and *p* = 0.04698 for Shannon and Chao1, respectively). Compared to the survival group, patients in the death group showed a more significant reduction in α diversity. Alpha-diversity did not differ between the two groups as expressed by Shannon or Chao1 diversity index for the gut microbiome (*p* = 0.90127 and *p* = 0.72816 for Shannon and Chao1, respectively). The beta-diversity of the lung and gut microbial composition did not significantly differ between the two groups (Figures 2B, C). OTU rank curves were generated to illustrate the microbial community structure. These OTU rank curves suggest that there were differences in the microbial community structure between two groups in both lung and intestine samples and the survival group had higher species richness and uniformity than the death group (Figures 2D, E). The relative abundance of species was mainly concentrated in 0.1–10%.

**Figure 3:**
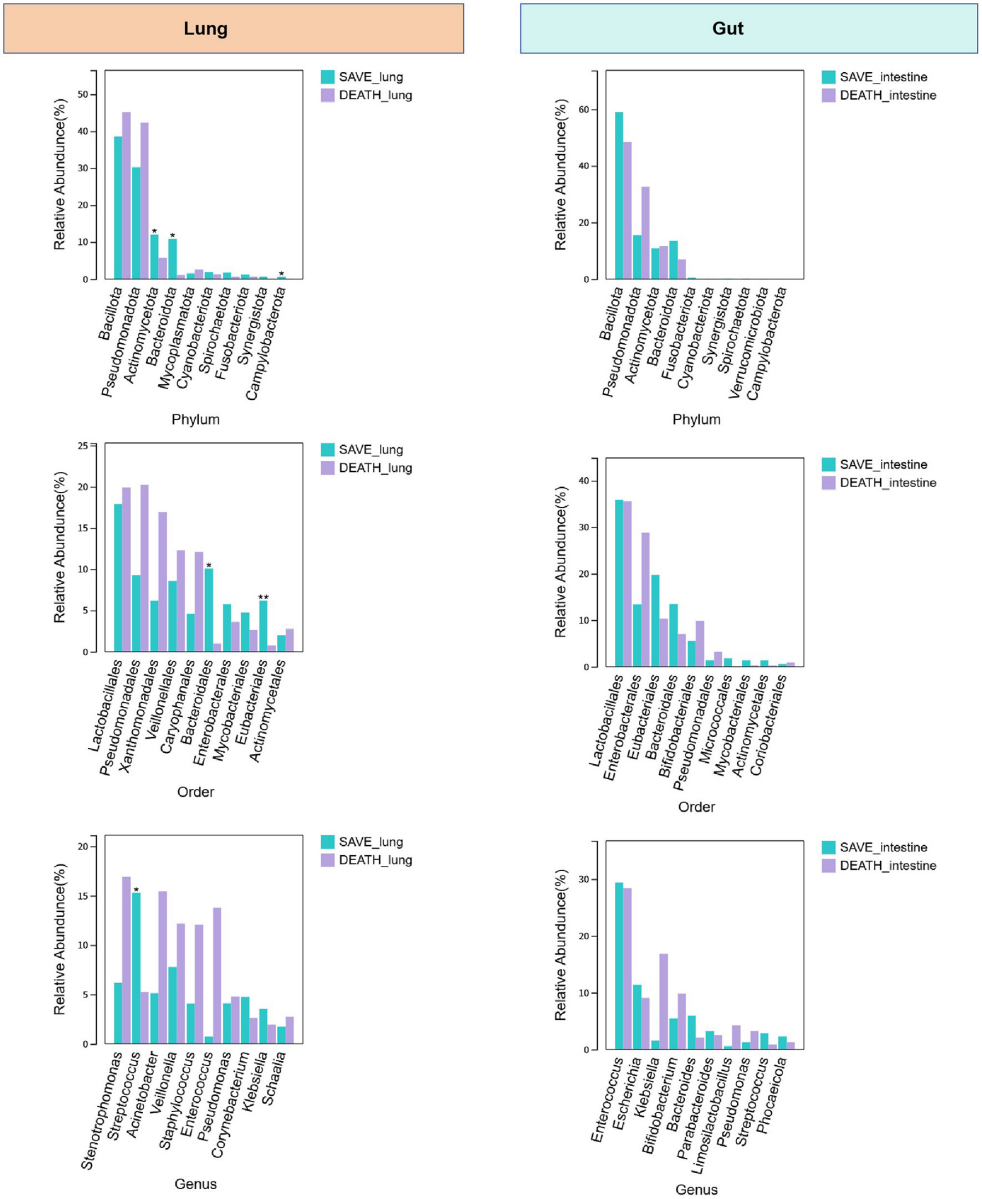
Average relative abundances of the predominant bacterial taxa of lung and gut at the phylum, order and genus levels between the death group and the survival group, with each color representing a taxon.

To illustrate the microbial community composition differences, we analyzed the relative abundance of microbes at phylum, order, and genus levels in lung and gut samples from survival and death groups (Figure 3). In respiratory microbiota landscapes, at the phylum level, the relative abundance of Bacteroidota, Actinomycetota and Campylobacterota in the survival group is higher than that in the death group; at the order level, the relative abundance of Bacteroidales and Eubacteriales in the survival group is higher than that in the death group; at the genus level, the relative abundance of Streptococcus in the survival group is higher than that in the death group. In gut microbiota landscapes, level taxa differed in abundance between groups, but the difference was not statistically significant. Collectively, these results indicated significant differences in microbial community structure between survival and death groups in both lung and gut ecosystems across multiple taxonomic levels, especially for respiratory microbiota landscapes.

### 3.3 LEfSe and UPGMA analysis of respiratory and intestinal flora between the death group and the survival group of SCAP patients

**Figure 4:**
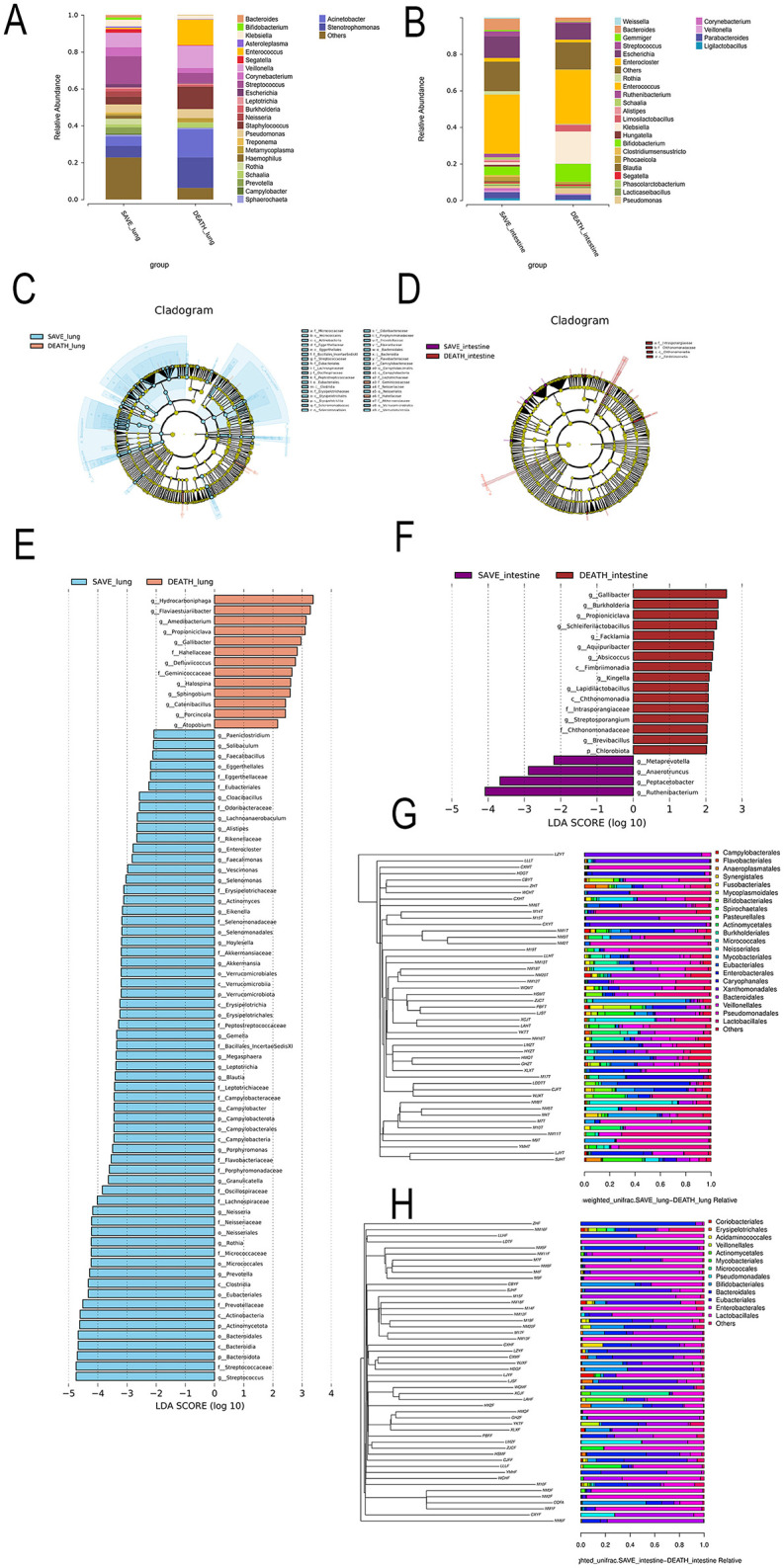
**(A)** lung and **(B)** gut microbial composition at genus level between the death group and the survival group of SCAP patients, with each color representing one genus. LEfSe cladogram for **(C)** respiratory flora and **(D)** intestinal flora between the death group and the survival group of SCAP patients. Different colors represent different groups, and nodes of different colors indicate microbial groups that play important roles in the group represented by that color. A colored circle represents a biomarker, and the legend in the upper right corner shows the name of the biomarker. Yellow nodes represent microbial taxa that do not play important roles in different groups. From the inside to the outside, each circle sequentially represents species at the phylum, class, order, family, and genus levels. Comparison of linear discriminant analysis (LDA) of **(E)** respiratory flora and **(F)** intestinal flora between the death group and the survival group of SCAP patients, which showed species with differences in LDA scores above the set point (2.0), i.e., statistically significant differences. UPGMA analysis of **(G)** respiratory flora and **(H)** intestinal flora between the death group and the survival group of SCAP patients.

Barplot analysis revealed that the lung and gut bacterial relative abundance were significantly decreased in the death group compared with the survival group (Figures 4A, B). LEfSe analysis could be used to further evaluate respiratory and intestinal flora between the death group and the survival group of SCAP patients. Figures 4C, D were the LEfSe cladogram. Compared with the death group, there were different respiratory microbiota in the survival group, which were Micrococcaceae, Micrococcales (a,b), Coriobacteriaceae, Coriobacteriales (d,e), Verrucomicrobiales, Verrucomicrobia (a8,a9), Neisseriaceae, Neisseriaceae (a4,a5), Erysipelotrichaceaeo, Erysipelotrichalesc, Erysipelotrichia (n,o,p), Selenomonadales, Selenimonas (q,r), Bacteroidales, Bacteroidaceae (w,x). Meanwhile, Geminicoccaceae, Hahellaceae (a3, a6) played an important role in the death group. However, as illustrated in Figure 4D for intestinal flora, Intrasporangiaceaeb (a), Chthonomonadaceaec, Chthonomonadiad (b,c), Fimbriimonadia (d) played an important role in the death group. In Figures 4E, F, LDA score was selected to more intuitively show the composition of different lung and gut microflora. Compared with the survival group in the respiratory flora, a total of 76 bacterial groups were significantly changed in the death group, including 63 down-regulated and 13 up-regulated bacteria groups. Hydrocarboniphagag, Flaviaestuariibacterg, Amedibacteriumg and Propioniciclava were enriched in the death group; Bacteroidalesc, Bacteroidiap, Bacteroidotaf, Streptococcaceaeg and Streptococcus were enriched in the survival group (Figure 4E). Besides, for intestinal flora, Gallibacterg, Burkholderiag, Propioniciclavag and Schleiferilactobacillus were enriched in the death group; Anaerotruncus, Peptacetobacter and Rutheniibacterium were enriched in the survival group (Figure 4F). On the basis of UPGMA analysis, we further demonstrated that the microbial communities were reduced in death group (Figures 4G, H). These findings suggested that the predominant beneficial bacteria decreased in death group and the disruption of microecological balance may be closely related to the prognosis of SCAP patients.

### 3.4 Correlation of microbiota features with clinical parameters

**Figure 5:**
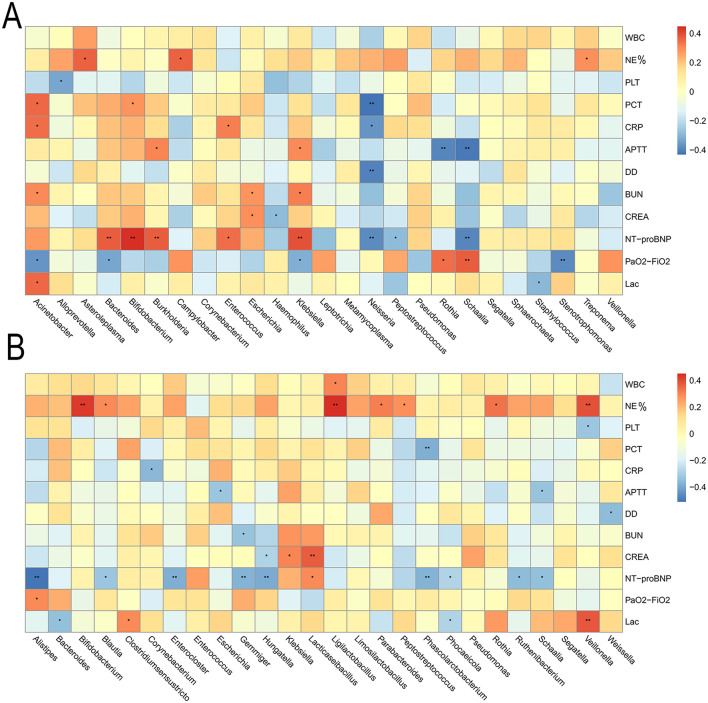
**(A)** Correlation between lung microbes and clinical parameters **(B)** Correlation between gut microbes and clinical parameters. Red bars indicate positive associations, while blue bars indicate negative associations. The color key indicates the association strength and direction in terms of the *t-*test. Pearson's correlation coefficient and *p-*value are used for plotting. Black asterisks indicate associations with *p-*value. WBC, white blood cell; PLT, platelet count; PCT, procalcitonin; CRP, C-reactive protein; APTT, activated partial thromboplastin time; NE%, neutrophilic granulocyte percentage; DD, D-dimer; SUN, Blood Urea Nitrogen; CREA, Creatinine; PaO_2_/FiO_2_, Oxygenation Index; Lac, Lactic Acid. ^*^p < 0.05, ^**^p < 0.01.

We ultimately tested whether identified key features of the microbiome were correlated with clinical parameters. As was shown in Figure 5A about correlation between lung microbes and clinical parameters, Asteroleplasma and Campylobacter were positively correlated with neutrophil percentage. Acinetobacter was positively correlated with PCT or CRP, while Neisseria was negatively correlated with PCT or CRP. These bacteria were closely involved in inflammatory responses. Schaalia was positively correlated with PaO_2_/FiO_2_, while Stenotrophomonas was negatively correlated with PaO_2_/FiO_2_. As was shown in Figure 5B about correlation between gut microbes and clinical parameters, Bifidobacterium, Ligilactobacillus and Veillonella were positively correlated with neutrophil percentage; Corynebacterium was positively correlated with CRP; Phascolarctobacterium was negatively correlated with PCT. Alistipes was positively correlated with PaO_2_/FiO_2_. Overall, these data suggested an association between microbiota composition and clinical parameters, predicting patient prognosis and disease severity.

## 4 Discussion

In this research, we investigated the pulmonary and intestinal microbiome of SCAP patients. To the best of our knowledge, this represents the first clinical investigation comparing lung and gut microbiota profiling between fatal and surviving SCAP patient groups. We aim to characterize compositional changes of these two sites and evaluate if the death patients present a different microbiota profiling.

In line with previous studies (Del Prete et al., 2025; Emonet et al., 2019; Enaud et al., 2023; Kritikos et al., 2025; Kyo et al., 2019; Schmitt et al., 2020; Zhou et al., 2023), the mortality patients or critically ill patients exhibit diminished alpha diversity of the lung microbiota, which may contribute to the pro-inflammatory responses observed in these patients. This aligns with the notion that diminished microbial diversity can exacerbate disease severity by altering host immune responses and microbial interactions (Del Prete et al., 2025). In our research, the death group in SCAP patients also had lower baseline alpha diversity of the lung microbiota compared with the survival group. This suggests that reduced microbial diversity may be linked to increased disease severity and mortality in respiratory conditions. Interestingly, the gut microbiota exhibited a similar trend, although the reduction in alpha diversity was less pronounced compared to the lung microbiota. The differential impact of SCAP on lung vs. gut microbiota alpha diversity observed in our study warrants further consideration. The more pronounced reduction in lung microbiota diversity in the death group, compared to the relatively preserved gut microbiota diversity, may reflect several mechanisms. First, the lung represents the primary site of infection and inflammation in SCAP, experiencing direct pathogen exposure and intense local immune responses that more dramatically reshape the microbial landscape. Second, the temporal dynamics of microbiota disruption may differ between compartments, with lung dysbiosis occurring more rapidly and severely in acute respiratory infections, while gut microbiota changes may require longer duration or more systemic effects to manifest significantly. Third, the gut microbiome's greater resilience and redundancy, given its higher baseline diversity and more stable environment, may buffer against acute inflammatory insults compared to the more vulnerable lung microbiome. These findings suggest that lung microbiota profiling may serve as a more sensitive prognostic marker than gut microbiota in the acute phase of SCAP, though the gut-lung axis likely remains important for disease pathogenesis through metabolic and immunomodulatory mechanisms. A study on critically ill patients revealed that early reductions in gut microbiota diversity were associated with increased mortality (Wozniak et al., 2024). This highlights a broader pattern where decreased microbial diversity, whether in the gut or lungs, correlates with poorer health outcomes. The study emphasized the need for early interventions to preserve microbiota diversity as a strategy to improve survival rates in critically ill patients. Collectively, these studies suggest that interventions aimed at preserving or restoring microbiota diversity could be beneficial in managing severe respiratory conditions and improving patient survival.

To identify the compositional changes in microbial heterogeneity most relevant to our study, we emphasized the dominance of lung predominant taxa (Bacteroidalesc, Streptococcus) in the survival group of SCAP patients and the dominance of gut predominant taxa (Anaerotruncus, Peptacetobacter, Rutheniibacterium) in the survival group of SCAP patients. The primary beneficial microbial populations were significantly reduced in the mortality cohort, suggesting that an imbalance in the microecological environment might be critically associated with the clinical outcomes of SCAP patients. These taxa are part of a complex microbial ecosystem that influences host health through various mechanisms, including immune modulation and metabolic interactions. Anaerotruncus, another key player in the gut microbiota, is known for its role in butyrate production, a SCFA with anti-inflammatory properties. The presence of butyrate-producing bacteria like Anaerotruncus is associated with maintaining intestinal barrier integrity and regulating immune responses, which are critical in the context of severe infections such as SCAP. The study on gut microbiota and autoimmune diseases underscores the importance of butyrate producers in maintaining immune homeostasis and preventing dysbiosis-related pathologies (Bhutta et al., 2024). This suggests that Anaerotruncus may contribute to the resilience of SCAP patients by supporting gut barrier function and reducing systemic inflammation. Peptacetobacter and Rutheniibacterium, although less studied, are emerging as important components of the gut microbiome with potential roles in metabolic and immune interactions. The study on gut microbial predictors of immunotherapy efficacy in non-small cell lung cancer patients highlights the predictive value of specific gut bacteria in treatment outcomes (Grenda et al., 2025). This suggests that Peptacetobacter and Rutheniibacterium might also serve as biomarkers for predicting SCAP patient outcomes, given their involvement in complex microbial networks that influence host metabolism and immune responses. The presence and abundance of lung and gut microbiota of SCAP patients appear to be linked with survival outcomes. These taxa may exert their beneficial effects through mechanisms involving SCFA production, immune modulation, and maintenance of gut barrier integrity. Further research is needed to elucidate the specific pathways through which these bacteria influence SCAP outcomes, potentially paving the way for microbiota-targeted therapies in managing severe infections. While the interplay between gut and lung microbiota—termed the gut-lung axis—has been extensively documented in the literature (Dumas et al., 2018; Enaud et al., 2020; Stavropoulou et al., 2020; Zhang et al., 2020), empirical research simultaneously examining both microbial environments remains remarkably scarce (Dickson et al., 2016; Wolff et al., 2021). During critical illness, the conventional microbial transmission pathways of the aerodigestive tract appear to undergo a fundamental reversal (Papazian et al., 2020). In normal physiological conditions, the oropharynx serves as the predominant microbial reservoir for both pulmonary and gastric ecosystems. However, in critically ill patients, this dynamic dramatically shifts, with the extensively proliferated gut microbiota emerging as the primary source of microbial colonization for the oropharynx and respiratory system. In severe community-acquired pneumonia, the gut-lung axis plays a particularly prominent role. Research indicates that the gut microbiome can modulate lung infection outcomes by influencing immune cell migration and inflammatory responses in the lungs (Ni et al., 2023; Schuijt et al., 2016). For instance, gut microbiome dysbiosis may lead to bacterial translocation in the lungs, thereby exacerbating acute respiratory distress syndrome (ARDS) (Dickson et al., 2016). Furthermore, fecal microbiota transplantation (FMT) has been shown to improve the prognosis of lung infections caused by Klebsiella pneumoniae, further supporting the critical importance of the gut-lung axis in pulmonary infections (Tang et al., 2024).

We sought for microbiota biomarkers capable of predicting clinical parameters in SCAP patients. In our study, we found that lung bacteria such as Asteroleplasma, Campylobacter and Acinetobacter and intestinal bacteria such as Bifidobacterium, Ligilactobacillus, Veillonella, and Corynebacterium were positively correlated with inflammatory markers PCT or CRP or neutrophil percentage. The relationship between specific bacterial taxa and inflammatory markers has been a subject of increasing interest, particularly in the context of lung and intestinal microbiota. This exploration is crucial for understanding the microbiota's role in systemic inflammation and its potential implications for disease management. The role of Bifidobacterium in modulating inflammation is well-documented. Bifidobacterium longum, for instance, has been shown to alleviate intestinal barrier damage and reduce inflammatory responses, which are often marked by elevated CRP levels (Li et al., 2024). This aligns with findings that Bifidobacterium can suppress gut inflammation caused by antibiotic disturbances, highlighting its potential to modulate inflammatory pathways (Ojima et al., 2020). Moreover, Bifidobacterium's ability to decrease colonic lipopolysaccharide concentrations suggests a mechanism by which it might reduce systemic inflammation, as lipopolysaccharides are known to trigger inflammatory responses (Rodes et al., 2013). These studies collectively underscore the anti-inflammatory potential of Bifidobacterium, particularly in conditions characterized by elevated CRP. In the context of lung microbiota, the association between airway bacteria and systemic inflammation markers such as CRP is also significant. Studies have shown that specific airway microbiota compositions are linked to systemic inflammation in conditions like non-small cell lung cancer, where CRP levels are used as an indicator of inflammation (Huang et al., 2025). Similarly, in chronic airway diseases such as bronchiectasis, the composition of the respiratory microbiome has been associated with varying levels of inflammatory cytokines, suggesting a direct link between lung microbiota and systemic inflammatory markers (Konovalovas et al., 2024). These findings highlight the potential role of lung bacteria, including Asteroleplasma, in influencing systemic inflammation. Furthermore, the interplay between gut and lung microbiota and their collective impact on systemic inflammation is evident in conditions such as chronic obstructive pulmonary disease (COPD). The gut-lung axis is increasingly recognized as a significant factor in inflammatory diseases, with studies indicating that gut microbiota can influence lung inflammation and vice versa (Chiu et al., 2021). This bidirectional relationship underscores the importance of considering both gut and lung microbiota in understanding and managing inflammatory conditions marked by elevated PCT and CRP levels. The correlation between microbial presence and the PaO2/FiO2 ratio is a critical area of investigation in understanding respiratory pathophysiology, particularly in conditions such as acute respiratory distress syndrome (ARDS), severe pneumonia and other forms of respiratory failure. Our study revealed that lung bacteria such as Schaalia and intestinal bacteria Alistipes were positively correlated with PaO_2_/FiO_2_, while lung bacteria such as Stenotrophomonas was negatively correlated with PaO_2_/FiO_2_. The positive correlation between Schaalia and the PaO2/FiO2 ratio suggests that the presence of this bacterium may be associated with better oxygenation status. This could be due to the potential role of Schaalia in modulating inflammatory responses or maintaining lung tissue integrity, thereby preserving lung function. The study by Vadi et al. highlights the importance of the PaO2/FiO2 ratio in classifying the severity of ARDS and its association with mortality, emphasizing the need for accurate assessment of this ratio in clinical settings (Vadi et al., 2024). Furthermore, the study by Alteration of Leukocyte Count et al. demonstrates the impact of systemic inflammation on pulmonary vascular permeability and oxygenation, which could be influenced by microbial presence (Johansson et al., 2015). Conversely, the negative correlation between Stenotrophomonas and the PaO2/FiO2 ratio indicates that this bacterium may contribute to impaired oxygenation. Stenotrophomonas is known for its multidrug resistance and association with nosocomial infections, which can exacerbate lung injury and inflammation, leading to a decrease in the PaO2/FiO2 ratio. The study by the Association between different indexations of extravascular lung water supports this notion that increased extravascular lung water, often a result of inflammation and infection, is associated with a lower PaO2/FiO2 ratio (Huber et al., 2014). Additionally, the study by the Influence of Hypercapnia and Atmospheric Pressure discusses how variations in the PaO2/FiO2 ratio can reflect underlying pathophysiological changes, including those induced by bacterial infections (Gilissen et al., 2022). The clinical implications of these findings are significant, as they suggest that monitoring the presence of specific bacteria could provide valuable insights into the respiratory and inflammatory status of patients. This could lead to more targeted therapeutic interventions aimed at modulating the microbial environment in the lungs and guts to improve patient outcomes.

We must acknowledge a few limitations to our study. First, this study consists of a small sample size which may influence the robustness of our findings regarding microbial features and further larger studies are yet required to better characterize the gut-lung axis of SCAP patients. Second, because of sample availability, we collected samples used for lung microbiota analysis were not exclusively bronchoalveolar lavage fluid, but also included sputum, which may have some influence on the description of the lung microbiota. Third, our research did not encompass metabolomic, transcriptomic, or proteomic investigations, nor did we explore the potential correlations between these molecular profiles and alterations in the lung and gut microbiome. Fourth, a limitation concerns the timing of antibiotic administration relative to sample collection. While we excluded patients who received antibiotics in the 30 days prior to enrollment, we did not systematically document whether emergency antibiotics were administered between hospital admission and sample collection (within 48 h). Fifth, our study's single time-point sampling design limits our ability to distinguish whether the observed microbiota alterations are causes or consequences of disease severity. The cross-sectional nature of our data precludes determination of temporal dynamics and causal relationships between microbiota composition and clinical outcomes. It remains unclear whether the reduced microbial diversity and altered community structure in the death group represent predisposing factors that contributed to worse outcomes, or alternatively, reflect secondary changes resulting from more severe disease, greater physiological stress, or differences in clinical management. Future research should focus on: Stricter protocols to ensure all samples are collected prior to any antibiotic administration; Longitudinal tracking of microbial changes during SCAP progression; Investigating the immunomodulatory mechanisms of identified bacterial taxa; Exploring potential therapeutic interventions targeting microbiota composition.

## Data Availability

The original contributions presented in the study are included in the article, further inquiries can be directed to the corresponding author/s.
